# Auditory Rehabilitation in Single-Sided-Deafened Patients after Surgery to the Cerebellopontine Angle for Vestibular Schwannoma: What Is the Patient’s Choice?

**DOI:** 10.3390/jcm13195967

**Published:** 2024-10-08

**Authors:** Margaux Loukine Bézé, Mathilde Puechmaille, Chloé Trillat, Antoine Barrat, Justine Bécaud, Nicolas Saroul, Toufic Khalil, Guillaume Coll, Thierry Mom

**Affiliations:** 1 Department of Otolaryngology Head Neck Surgery, University Hospital Center, Hospital Gabriel Montpied, 58 rue Montalembert, 63000 Clermont-Ferrand, France; mloukine@chu-clermontferrand.fr (M.L.B.); mpuechmaille@chu-clermontferrand.fr (M.P.); ctrillat@chu-clermontferrand.fr (C.T.); abarrat@chu-clermontferrand.fr (A.B.); jbecaud@chu-clermontferrand.fr (J.B.); nsaroul@chu-clermontferrand.fr (N.S.); 2 Mixt Unit of Research (UMR) 1107, National Institute of Health and Medical Research (INSERM), University of Clermont Auvergne (UCA), 63000 Clermont-Ferrand, France; 3 Department of Neurosurgery, University Hospital Center, Hospital Gabriel Montpied, 58 rue Montalembert, 63000 Clermont-Ferrand, France; tkhalil@chu-clermontferrand.fr (T.K.); gcoll@chu-clermontferrand.fr (G.C.)

**Keywords:** vestibular schwannoma, single-sided deafness, bone-anchored hearing aid, contralateral routing of signal (CROS) hearing aid, hearing in noise test (HINT)

## Abstract

**Background**: Surgical resection of vestibular schwannomas (VS) can be responsible for single-sided deafness (SSD). Hearing restoration can be a challenge both for the otolaryngologist and the patient. **Patients and Methods**: In a retrospective series, we analyzed the charts of SSD patients operated on for VS from 2005–2021, checking which type of hearing rehabilitation was chosen. All patients who wanted a hearing restoration underwent a hearing in noise test (HINT) in a stereo auditorium with and without a bone-anchored hearing device (BAHD) worn with a headband on the deaf side. Then, they had a preimplantation one-month trial with the BAHD at home vs. contralateral routing of signal (CROS) or BiCROS (with contralateral signal amplification) hearing aids (HAs). **Results**: Among 52 charts of the included adult SSD patients, only 29 (56%) eventually chose a hearing rehabilitation device (14 BAHD). Only one BAHD patient required a device explantation for skin complications, but then asked for reimplantation. Another one swapped the BAHD for HAs 2.5 years after. Two patients only occasionally used their BAHD with a headband. Nine patients preferred HAs, mainly BiCROS. Their contralateral hearing was significantly less than BAHD patients (*p* < 0.05), and only three used their HAs every day. **Conclusions**: Hearing rehabilitation in SSD patients after VS surgical resection is chosen in about 50% of cases. In complement of HINT, a real-life comparative hearing trial helps patients chose the best device, with good long-term results when a BAHD is chosen. HAs are preferred when contralateral hearing is altered but are not always worn.

## 1. Introduction

Trends in the management of vestibular schwannomas (VS) have shifted over the last two decades from total removal regardless of the functional result to functional preservation [1]. While postoperative facial nerve function is the main concern of patients scheduled for surgical removal of VS, postoperative hearing is increasingly considered [2,3,4]. Despite a lot of advances in intraoperative hearing monitoring, using auditory brainstem response (ABR) [5], distortion-product otoacoustic emissions (DPOAEs) [6,7], electrocochleography (EcoG) [5,8], electroneuronography and other techniques [9], the rate of hearing preservation in tumors larger than KOOS classification stage II remains poor, most often <50% [10,11]. This means that at least 50% of patients end up with single-sided deafness (SSD) on the operated side. We wanted to know the rate of SSD patients seeking auditory rehabilitation and, if so, the type of device chosen, namely conventional hearing aids (HA) on the hearing side, HAs with contralateral routing-of-signal (CROS) or bilateral CROS (BiCROS) or finally, a bone-anchored hearing aid (BAHD).

In the case of postoperative SSD, one can guess that patients are highly disabled in everyday life in understanding their surroundings, especially in noise, at least for those who had useful hearing on the operated side pre-operation. One of the more difficult situations is known to be due to the head shadow, that is, when the speaker is placed on the deaf side [12].

Cochlear implantation can have good results, but only if the nerve has been preserved during surgery, especially regarding sound localization, speech recognition in noise, tinnitus control [4], binaural functionality and quality of life [13,14,15]. However, it is of recent use, and the anatomical preservation of the cochlear nerve remains challenging in cerebellopontine angle (CPA) surgery. BAHD and HAs are less invasive devices, and they can compensate for the head shadow effect and have been shown to improve quality of life [16]. Moreover, BiCROS devices can help reduce tinnitus [17]. Despite a pseudo-stereophony, BAHD allows discrimination in noise [3].

Herein, we reported our series of patients operated on for VS with a postoperative SSD from 2005 to 2021. In this retrospective analysis, we sorted patients through their choice of hearing rehabilitation and checked whether they still used their device in the long follow-up. The aim was to help them choose the best auditory rehabilitation after such a heavy CPA surgical procedure.

## 2. Patients and Methods

All charts of patients operated on for VS from 2005 to 2021 with a postoperative SSD in our institution were collected. We looked at the tumor staging using the KOOS classification, preoperative hearing, postoperative hearing testing and finally, the choice of hearing rehabilitation. Patients were sorted into three groups depending on the auditory rehabilitation. Group A chose a BAHD, Group B chose HAs and Group C chose no auditory rehabilitation.

They were operated on by an otoneurosurgical team consisting of an otolaryngologist surgeon and a neurosurgeon. Patients were operated on through a retrosigmoid route or a translabyrinthine approach. In some cases, intraoperative monitoring was carried out using EcoG or DPOAEs, as already reported [6,8].

**Figure 1:**
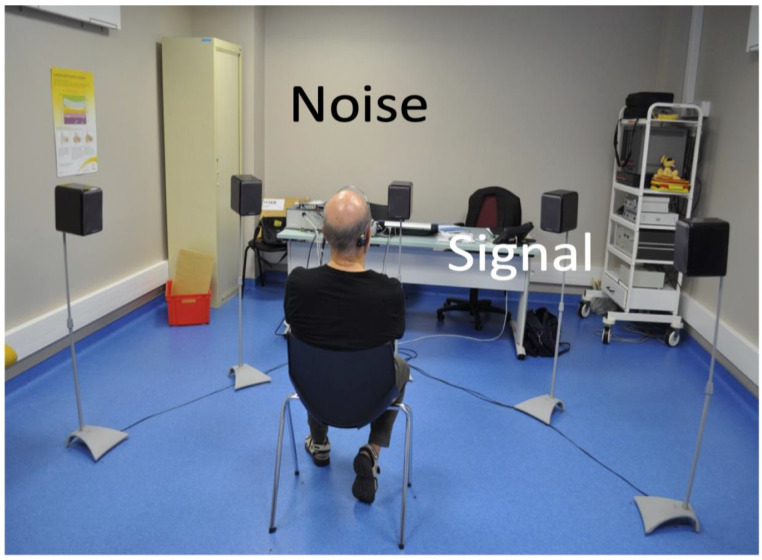
Simplified HINT for assessment of the head shadow effect. Here, the right ear is deaf. The noise comes from the front loudspeaker while the signal (sentences) is addressed to the deaf ear. The BAHD is worn directly on the right side on a percutaneous abutment in this patient. This test allows for comparisons of speech recognition scores and thresholds with and without BAHD.

The SSD patients who wanted to test a hearing rehabilitation device all had tonal and speech audiometry in silence in a soundproof booth six to twelve months after surgery in order to test their contralateral hearing. Their contralateral pure tone average (PTA) was built up from 250 Hz to 8 kHz by octave steps. Then, they underwent auditory battery testing in a stereo auditorium (BST) with five loudspeakers arranged in a semi-circle in front of the patient, as already reported [18]. A distance of one meter was respected between the head of the patient and each loudspeaker. Their performance of speech in noise was assessed using dissyllabic word lists, addressed to the deaf ear in the stereo auditorium open-field setting, in noise. White band noise was delivered through the frontal loudspeaker at 60 dB SPL in order to test the head shadow effect with a signal-to-noise ratio (S/N) of 0, 5 and 10, that is, a simplified Hearing in Noise test (HINT-Figure 1). Then, the same test was achieved with a BAHD worn with a soft band on the deaf side. Localization performance with and without the BAHD was assessed in the first period of this series and has already been published [18]. Because the localization performance in SSD patients proved to be poor with a BAHD, even one year later (12), we thus stopped testing it in these patients a few years ago. After completing this BST, or this simplified HINT, patients were oriented to an HA specialist who let them test the benefit of the BAHD vs. HAs in real-life conditions. CROS HAs were tried when contralateral hearing was normal or subnormal and BiCROS were tried when contralateral hearing could also benefit from amplification. Patients tried each type of hearing device for one month before making their choice.

Patients who chose to have BAHD were all operated on under local anesthesia. Nine of them had a “punch and drill” procedure following the latest manufacturer’s recommendation (Cochlear LTD^®^, Sydney, Australia). Briefly, with a 5 mm skin biopsy punch, skin and soft tissue were removed at the selected site of implantation behind the ear. Then, the first 3 mm deep drilling was achieved with a 2 mm guide drill followed by a 4 mm one after checking that the dura was not exposed. A bony well was then rimmed out with a dedicated widening drill at a 2000 r/s. Finally, in a one-step procedure, an auto-tapped BAHD implant with its abutment on was slowly inserted at a torque of 40 N/cm. One patient had a linear incision following the Nijmegen technique [19]. The access to the bony site of implantation was achieved through a 2.5 cm long linear incision. The BAHD insertion was then similar to the punch and drill technique and was achieved right in the middle of the skin incision. Two patients had a C-shaped skin incision with skin flap thinning and soft tissue resection, as recommended earlier. The BAHD was then bony implanted and the skin at the center of the skin flap was crossed out through a minimal incision.

We retrospectively verified the patients’ choice for hearing rehabilitation after this postoperative-specific hearing testing. For the patients of Group A who chose a BAHD rehabilitation, we checked the type of surgical procedure, the rate of major complications (i.e., ending in change or removal of the BAHD) and the actual long-term use of the BAHD by phone call. Patients were asked whether they used their BAHD all day or just in certain circumstances such as in groups, or while watching TV. For patients of Group B who chose HAs, we checked whether they swapped their HAs for a BAHD.

Preoperative auditory performance was verified for both ears, allowing us to grade them according to the American Academy of Otolaryngology Head and Neck Surgery (AAO-HNS) hearing classification system [20]. The duration of hearing loss on the operated side before the surgical procedure to the CPA was collected; contralateral hearing was also collected.

Comparisons of contralateral hearing pure tone average (PTA) between groups used an ANOVA with a post-hoc Scheffé test. Comparisons of the average speech performance at the three S/N ratios were compared with and without the BAHD using a McNemar test. Other comparisons were performed with ANOVA, chi-square test or Fischer’s exact test depending on the type of comparisons and the number of data points. A *p*-value ≤ 0.05 was considered statistically significant.

## 3. Results

Patients’ characteristics are described in Table 1.

A total of 52 SSD patients’ charts were included in the analysis.

As seen in the table, most patients had big tumors (≥Koos grade III). In each group, only three patients had a tumor less than 2 cm (<Koos grade III). There was no difference in tumor size between groups.

Twenty-nine (56%) of them preferred having no auditory rehabilitation, feeling an eventual low benefit of hearing rehabilitation regardless of the type of hearing device, or were reluctant to any type of additional medical intervention after their skull base surgery. Fourteen patients (27% of all patients; 61% of patients who chose a hearing device) chose a BAHD and nine chose HAs (17% of all patients; 39% of patients who chose a hearing device).

Auditory benefit was objectively noted in the HINT for all patients who chose a BAHD except one.

All but one patient improved their average speech understanding in noise during the HINT (McNemar test, *p* < 0.01). Unexpectedly, the patient who did not improve chose the BAHD after achieving the one-month real-life trial. The gap between the CPA surgery and BAHD implantation was on average 21 ± 14 months. Two patients preferred not to be implanted, since they occasionally wear their BAHD on a headband (Soundarc^®^) only when they are in groups.

Among all patients who chose an osseointegrated BAHD, only one (8.3%) asked for implant removal 31 months later. Another patient out of those who had an osseointegrated implant (8.3%) required a change of the abutment of the device because of skin covering 37 months after implantation, but he had no early complications. A total of 12 (86%) patients were still using their BAHD every day on the day of the phone call, that is, after a mean follow-up of 53 ± 50 months.

**Figure 2:**
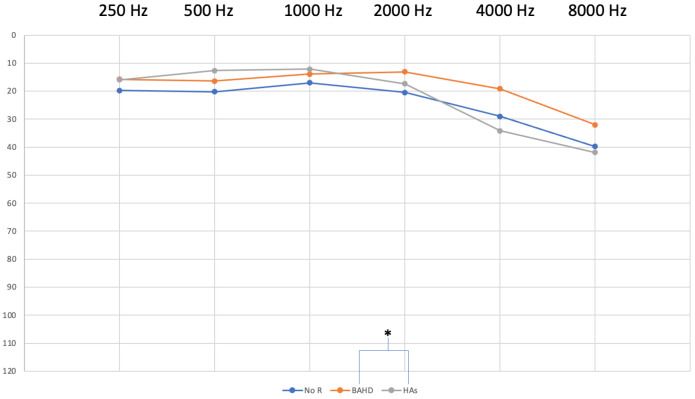
Average of contralateral hearing. The PTA was significantly better for the BAHD group over group B (HAs) (* ANOVA, post-hoc Scheffé test, *p* < 0.05). NoR: No hearing rehabilitation (Group C).

As far as HAs are concerned, 89% (8 out of 9) of patients chose a BiCROS system, the last one using a classical CROS system. Contralateral hearing was better on average in patients who chose a BAHD at 17 ± 8 dB HL, vs. the two other groups (ANOVA and post-hoc Scheffé test, *p* < 0.05). (Figure 2).

Patients who chose HAs had a contralateral PTA at 23 ± 12 dB HL (Figure 2), and patients of Group C who refused any type of hearing rehabilitation had a contralateral hearing at 22 ± 11 dB HL (Figure 2).

The difference was especially marked at 2 kHz and above. No patient who chose HAs swapped their devices for a BAHD. Only three patients who chose HAs wore their devices every day. Two patients did not use their HAs anymore on the day of the phone call, and the remaining four used them occasionally, either in groups or while watching TV.

The AAO-HNS hearing classification system was used to categorize all patients based on their auditory performance (Table 1) [20]. In this series, patients with grade C or D tumors, regardless of their group, never had HAs before the surgical procedure to the CPA. this means they did not use their impaired ear in the preoperative period. These ears were considered here as non-useful ones. Preoperatively, the rate of poor hearing ears (grades C and D) and good hearing ears (grades A and B) did not differ between groups (chi-square = 1.97, degree of freedom = 2, *p* > 0.3).

Concerning the duration of deafness in patients with one non-useful ear, there was a statistical difference between the groups, with a shorter duration of deafness of 15 ± 15 months in the BAHD group (ANOVA, *p* < 0.025) compared to 57 ± 59 months and 44 ± 51 months in the HAs and NoR groups, respectively.

## 4. Discussion

Very few papers reporting on SSD patient hearing rehabilitation after VS removal are available [21]. We report our series with a long-term follow-up (on average 5 years), mainly showing that only half of these patients choose hearing rehabilitation; after comparative testing of HAs vs. BAHD, most prefer a BAHD on the deaf side, with great long-term satisfaction. No such long-term series on SSD patient hearing rehabilitation after CPA surgery for VS, offering a comparative trial between hearing devices, has been reported yet to the best of our knowledge. Boucek et al. reported good results with a BAHA in such patients, but their patients did not try HAs before decision making [21]. We not only confirm that BAHAs are effective in SSD patients after VS removal, but that it is also likely preferred to HAs. In actuality, after a one-month preimplantation trial, 61% of patients who wanted hearing rehabilitation chose BAHA over HAs.

SSD is a real handicap in everyday life. Patients who have to be treated for VS from our experience are more and more demanding of hearing rehabilitation after surgery. They are all clearly informed that when hearing is lost on one side, not only does sound localization become poorer, but so does hearing noise, that is, hearing in everyday life conditions. Although most of the central auditory function is compromised in SSD, the head shadow effect is the prominent handicap felt by patients [22]. This can be revealed by audiometric testing in noise. Several strategies are available nowadays. For years, we chose a very simple simplified HINT with noise in the front and the word lists addressed on the deaf side. Patients can immediately feel the correction brought by the BAHD. This modified HINT is a very quick procedure with only six word lists addressed to the deaf side in noise with and without BAHD; thus, it is a well-tolerated procedure by the specific patients who underwent heavy CPA surgery. This modified HINT proved to be sufficient to clearly reveal the head shadow effect. To be sure they would choose a BAHD, a trial vs. BiCROS or CROS HAs at home in everyday life helped them ascertain their choice. Indeed, SSD can be rehabilitated by different means, not only with BAHD but also with HAs, and in some cases cochlear implants. Herein, we confirmed that we can correctly select patients who can have a satisfactory functional result with a BAHD, which is in accordance with previous reports [18]. One interesting result is that most patients of our series who preferred a BAHD were still using it many years after, with 13 out of 14 of our series (83%) still using it every day five years after. Herein, only one patient asked for BAHD removal and a swap for a BiCROS HA. In contrast, another one who had a skin complication nevertheless asked for a new BAHD, confirming the highly appreciated hearing benefit he felt from his BAHD. He was the only case (about 8%) of severe long-term complications; a similar case had already been reported [21,23]. The surgical technique used was a “punch and drill” technique, with no immediate complication but a later one with skin covering. This ratio is in accordance with what has already been reported [22]. Two patients had a linear incision with no complications. BAHD was easily implanted in the incision, and no difference has been shown to date in the literature between implantation in or out of the skin incision regarding the rate of complication [19]. The patient who chose a BAHD despite a bad HINT result reveals that the real-life trial is mandatory. It seems that the HINT cannot measure all the potential benefits of a BAHD or BiCROS HAs. Comparative trials are very informative, and we completely agree with the recent publication reported by A.W. Wendrich et al. on pre-hearing rehabilitation trials [16].

It is noteworthy that patients who chose a BAHD had significantly shorter preoperative hearing loss periods of 15 ± 15 months, while HAs patients and NoR patients had a longer preoperative hearing loss duration of approximately five and four years, respectively. We can recall BAHD patients used their auditory devices whereas others used their HAs on some occasions, such as in groups or while watching TV. This may suggest that in case of recent hearing loss, BAHD may provide better hearing comfort than HAs in SSD patients.

Patients who preferred HAs over BAHD had also significantly poorer contralateral PTA. This is not surprising since BiCROS HAs can better improve hearing in the good ear in SSD patients compared to BAHD. Surprisingly, among the patients who chose HAs, only one-third still used them every day when we called them, which was about 8 years later on average. This compares unfavorably to BAHD with only two patients using it occasionally with a headband. These patients, who only have mild or moderate hearing loss in their good ear, may have difficulty accepting wearing a hearing aid on this ear.

Another important result is that many SSD patients in this series (over 50%) did not want any hearing rehabilitation. This has already been reported [24,25,26], and preimplantation testing is also mandatory for this reason. The reasons for hearing rehabilitation refusal are likely numerous, and without any doubt depend on patients’ everyday lives, who would rather live lonely or, in contrast, often be in groups and meetings. The decision to refuse any hearing rehabilitation may also rely on the patient’s will to avoid any type of additional surgical procedure or even any hearing investigation after a heavy surgical procedure to the CPA. This last reason could explain the difference with the series of SSD patients recently reported by A.W. Wendrich et al., with a majority of patients preferring hearing rehabilitation [16].

Cochlear implantation has recently been proposed to rehabilitate hearing in SSD patients. It has been reported in some cases after VS removal [4,13,14]. The challenge is to check the integrity of the cochlear nerve after VS removal, which is not easy to achieve and may require placing a cochlear implant within the cochlea during VS resection, taking the risk of not using it if the cochlear nerve cannot be preserved [27]. In addition, cochlear implants in SSD patients are not accepted in all countries. For example, in France, a cochlear implant is only reimbursed in SSD patients with disabling tinnitus [28]. Based on a controlled trial of BAHD vs. cochlear implant vs. CROS HAs, Marx et al. could only conclude the cochlear implant’s superiority in the case of disabling tinnitus [29]. Other teams have reported good hearing results with cochlear implants after unilateral VS removal, such as Plontke et al. in the case of intracochlear schwannomas [30]. In our series, in which analysis ends in 2020, we did not propose this cochlear implant alternative to our patients.

We acknowledge that our study has some limitations. It is based on a retrospective series; however, it bears the advantage of a long follow-up (5–8 years). It is not a big series, with only 52 charts analyzed, but, to our knowledge, few clinical series focused on hearing rehabilitation in SSD patients after VS removal are available and they are not larger (e.g., Boucek et al. [21]).

## 5. Conclusions

Hearing rehabilitation is wanted by no more than half of SSD patients after VS removal. A precise preoperative hearing assessment focused on the head shadow effect is usefully completed by a comparative preimplantation trial at home for BAHD vs. CROS or BiCROS HAs to choose the adequate rehabilitation with a high rate of satisfaction. BAHD is likely a good long-term solution when contralateral hearing is good and is the preferred hearing rehabilitation means in cases of recent hearing loss, while BiCROS HAs seem preferable whenever contralateral hearing requires a hearing aid, even though they are not worn all day long.

## Figures and Tables

**Table 1:** Characteristics of patients. N/A; Non Applicable.

Patient	Age	Sex	KOOS	Preoperative PTA	Duration of Hearing Loss before Surgery	Follow-Up (Months)	Type of HA	Device Use	Complications (for BAHD)
BN-01	52	F	IV	21.25	0	43	BAHD	every day	1
BS-02	65	F	IV	28.75	8	91	BAHD	every day	0
DI-03	44	F	II	15	0	67	BAHD	every day	0
CJ-04	24	F	IV	cophose	4	122	BAHD	every day	0
AC-05	60	F	I	cophose	15	210	BAHD	every day	0
DA-06	57	F	III	20	0	35	BAHD	every day	0
GM-07	65	F	IV	68.75	36	90	BAHD	every day	0
GA-08	60	M	IV	26.25	0	40	BAHD	every day	0
HM-09	58	F	IV	40	6	62	BAHD	every day	N/A-headband (Soundarc^®^)
HN-10	49	F	IV	38.75	48	44	BAHD	every day	N/A-headband (Soundarc^®^)
PA-11	40	F	IV	6.25	0	59	BAHD	every day	0
SC-12	31	F	IV	20	0	167	BAHD	every day	0
SB-13	68	F	IV	48.75	0	97	BAHD	every day	0
TC-19	67	F	II	48.75	84	61	BAHD	occasionally	1
BD-01	66	M	IV	cophose	6	87	BICROSS	occasionally	N/A
CG-02	84	M	IV	66.25	120	92	BICROSS	occasionally	N/A
DJ-03	74	F	II	75	7	98	BICROSS	no use	N/A
DF-04	54	M	IV	18.75	0	84	CROSS	every day	N/A
JA-08	38	F	II	12.5	0	74	BICROSS	occasionally	N/A
LB-10	64	F	II	90	120	170	BICROSS	every day	N/A
MM-12	58	M	IV	35	13	107	BICROSS	no use	N/A
SJ-14	75	M	IV	80	96	80	BICROSS	every day	N/A
SI-15	66	F	IV	17.5	0	104	BICROSS	N/A	N/A
BP-01	66	M	IV	40	4	86	no device	N/A	N/A
BD-02	70	F	III	35	18	109	no device	N/A	N/A
CF-03	53	M	III	30	21	121	no device	N/A	N/A
RH-04	77	F	IV	cophose	180	49	no device	N/A	N/A
CB-05	76	M	IV	86.25		109	no device	N/A	N/A
CH-06	61	M	IV	cophose	84	104	no device	N/A	N/A
DM-08	60	F	III	70	11	89	no device	N/A	N/A
DJ-09	85	F	II	43.75	12	182	no device	N/A	N/A
FP-10	59	M	II	cophose	36	49	no device	N/A	N/A
FM-11	67	F	IV	cophose	72	94	no device	N/A	N/A
GP-12	51	M	III	cophose	8	121	no device	N/A	N/A
JC-15	71	F	IV	72.5	12	103	no device	N/A	N/A
JC-16	57	F	III	62.5	40	99	no device	N/A	N/A
LA-17	85	F	IV		156	111	no device	N/A	N/A
LO-16	62	M	IV	43.75	11	91	no device	N/A	N/A
LV-18	62	F	IV	cophose		55	no device	N/A	N/A
MC-20	77	F	IV	66.25	7	108	no device	N/A	N/A
MR-21	56	F	III	cophose	24	110	no device	N/A	N/A
NF-23	50	F	IV	22.5	0	71	no device	N/A	N/A
OM-24	75	M	II	46.25	60	207	no device	N/A	N/A
PF-15	35	F	IV	37.5	18	60	no device	N/A	N/A
RT-26	69	F	IV	53.75	12	88	no device	N/A	N/A
SM-28	84	F	IV	cophose	22	66	no device	N/A	N/A
SJ-29	76	M	IV	61.25	24	95	no device	N/A	N/A
TV-30	DCD	F	IV	60	10	97	no device	N/A	N/A
VM-32	76	F	IV		0	89	no device	N/A	N/A
VG-33	67	M	IV	cophose	60	73	no device	N/A	N/A
DC-34	67	F	IV	56.25	4	97	no device	N/A	N/A
DP-35	63	M	IV	25	120	38	no device	N/A	N/A

## Data Availability

Our analytic data are stored confidentially in our institution and protected for ethical reasons, while the averages are shown in the text. For further inquiries or specific data requests, can contact the corresponding author directly.
